# Outreach acute care for nursing homes: an observational study on the quality and cost-effectiveness of the Mobile Hospital

**DOI:** 10.1093/ageing/afae287

**Published:** 2025-01-07

**Authors:** Kontunen Perttu, Leppänen Roope, Linna Miika, Castrén Maaret, Torkki Paulus

**Affiliations:** Department of Public Health, University of Helsinki, Helsinki, Uusimaa, Finland; Hospital Services, Western Uusimaa County Wellbeing Services, Espoo, Uusimaa, Finland; Department of Health and Social Management, Faculty of Social Sciences and Business Studies, University of Eastern Finland, P.O. Box 1627, FI-70211, Kuopio, Pohjois-Savo, Finland; Helsinki University Central Hospital—Emergency Medicine, University of Helsinki and Department of Emergency Medicine and Services, P.O. Box 340, FI-00290, Helsinki, Finland; Department of Public Health, University of Helsinki, Helsinki, Uusimaa, Finland; Department of Industrial Engineering and Management, Aalto University, P.O. Box 11000, FI-00076 AALTO, Espoo, Uusimaa, Finland

**Keywords:** acute care, nursing home, emergency department, emergency medical services, outreach, older people

## Abstract

**Background:**

The global trend of emergency department (ED) crowding can be mitigated with outreach care. The Mobile Hospital is an outreach acute care service in Espoo, Finland. This study describes the results of the Mobile Hospital intervention to nursing homes in a pre–post study setting with benchmarking validation data.

**Methods:**

We compared Emergency Medical Services (EMS) missions, ED visits, hospitalisations and their estimated costs from two 6-month periods in 2018–2019 (1325 nursing home beds). Benchmarking control data for ED visits were obtained from health records of the 10 largest Finnish cities.

**Results:**

The number of EMS missions to nursing homes decreased by 16% (720 vs 604), ED visits decreased by 22% (801 vs 622), there was no significant difference in specialised inpatient episodes (178 vs 162) and primary hospital inpatient episodes were fewer (285 vs 178, decreased 38%). Annual estimated savings per resident were 686 euros (decreased 14%). Annual estimated total savings were 934 908 euros. In the benchmarking analysis, the number of ED visits and acute hospitalisations amongst the older population decreased in Espoo, while in the other cities it increased.

**Conclusions:**

The Mobile Hospital seems to reduce nursing home residents’ ED visits, hospitalisations and overall costs. Advance care planning and on-call physician telephone consultations may be useful components of the service.

Implications to practice: This study adds to the growing evidence that outreach care to nursing homes is cost-effective in suburban areas with universal healthcare funding, at least as part of other developments in the acute care pathway.

## Key Points

- The Mobile Hospital addresses acute complaints of nursing home patients.
- The Mobile Hospital seems to reduce Emergency Department visits and hospitalisations.
- This service seems to lower healthcare costs compared to traditional Emergency Medical Services and Emergency Department visits.

## Background

Emergency Department (ED) crowding is a global phenomenon that challenges the healthcare system [1] and new ways to deliver acute care to patients are warranted. The Emergency Medical Services (EMS) is well equipped to manage the most critical patients, especially those requiring intensive interventions. These missions frequently result in the patient being transported to the ED for further treatment. Development of point-of-care (POC) testing, communication equipment and other novel remote access healthcare innovations enable acute care bedside. This outreach care is especially suitable to residents living in nursing homes—they are usually frail [2] and in most cases do not benefit fully from hospital-grade diagnostics and treatment because of burden from transfers and the risks and strain from the possible treatment. In most cases, a focus on quality of life is preferred. A recent systematic review showed that outreach service to nursing homes might reduce ED visits, hospitalisations and costs, at least when preventive measures are a part of the service model [3].

Promising results were seen in Danish and British studies on outreach units to nursing homes, and acute complaints normally requiring ED visit seemed to be mostly treatable on-site [4, 5]. Hutchinson *et al.* investigated an outreach unit [6] which reduced acute hospitalisations. A systematic review identified problems in nursing home acute care [7], which could possibly be addressed by an outreach care unit [3].

An example of a novel mobile outreach acute care service is the Mobile Hospital, an outreach unit operating in Espoo, Finland, a city of ~290 000 residents. The Mobile Hospital began in March 2019 and is staffed 24/7 by a specially trained nurse. The Mobile Hospital responds to acute consultation calls from nursing homes and, when nursing home care alone is insufficient or the situation requires further assessment, a nurse travels to the patient in a specially equipped sports utility vehicle. This vehicle includes a custom metallic chassis designed to hold medicine boxes, fluids and POC testing equipment, enabling the Mobile Hospital to provide on-site acute treatments such as antibiotics, POC testing and palliative care. The Mobile Hospital and EMS have been described in more detail in an earlier study [8].

In this study, we describe the results of the initiation of the Mobile Hospital in a pre–post study setting. To reduce the risk of confounding, we used also benchmarking validation data from Finland’s 10 largest cities to assess concurrent changes in ED visits amongst older population (people >75 years).

## Methods

### Objectives

Our primary objective was to examine the quality and cost-effectiveness of the Mobile Hospital intervention by comparing registry data from 2018 and 2019 six-month study periods. Quality was assessed using the dimensions outlined in the Institute of Medicine (IOM) definition, which includes effectiveness, timeliness and safety of the intervention [9]. In this study, quality is also defined in terms of process times (throughput times), readmissions and mortality rates.

### Setting of the study

We studied the Mobile Hospital intervention by

(1) Analysing the changes in process, outcomes and cost measures before and after the intervention (2018 six-month pre-period vs 2019 six-month post period), and

(2) Comparing the number of ED visits and acute hospitalisations amongst people >75 years in Espoo with the other biggest cities in Finland. This allowed a benchmarking-controlled study setting to validate the findings; the methodology is discussed in detail in an article by Malmivaara [10]. In this benchmarking-controlled setting, the results of the intervention setting (e.g. Emergency Services) were compared with the results of similar control settings to assess the effect.

In the pre–post setting, the primary outcome measures were the numbers of EMS missions, ED visits, specialised hospital episodes due to acute reasons = hospitalisation from the ED (not included elective episodes) and acute primary hospital episodes—the patient is usually transferred there to receive simpler treatment or when the diagnosis is clear and no other specialised treatments are planned or palliative treatment is assigned. We also reviewed the Mobile Hospital missions and total cost. From these data and list prices of EMS missions, ED visits and hospital days, we estimated the costs. As a relative safety marker, we compared mortality rates and 24-h ED readmission rates.

Cost-effectiveness was evaluated as cost per resident in nursing home and as cost per EMS (plus Mobile Hospital) patient.

The Mobile Hospital was planned and initiated when the study process began; therefore, we did not attend prospective collection of data or cluster-randomised study setting.

### Recruitment and data collection

We collected the data from electronic health records from city of Espoo (LifeCare by TietoEvry Oyj) and Helsinki University hospital (HUS) (Uranus, Merlot Medi by CGI Oy). The data were merged and pseudonymised by HUS Data authority. We received aggregate inpatient data from primary hospital ward and therefore the length of stay in primary hospital ward is based on average length of stay. We collected the benchmarking validation data from the National Registry of Hospital Visits and Inpatient episodes (HILMO) managed by the Institute of Health and Welfare (THL). We selected the 10 biggest cities in Finland: Helsinki, Kouvola, Kuopio, Lahti, Oulu, Pori, Tampere, Turku and Vantaa. The data from the City of Jyväskylä were not available. We used Strengthening the Reporting of Observational studies in Epidemiology (STROBE) checklist to ensure comprehensive reporting of the study [11]; the checklist is included as a supplementary table (see Appendix 2 in the Supplementary Data section).

### Data analyses

SPSS and Excel were used with χ^2^, *t*-test and Mann–Whitney *U* statistical tests as appropriate. We used Large language model ChatGPT 4 (OpenAI) to help proofread and present the results. The cost of acute care was calculated by multiplying the number of contacts with the Mobile Hospital, EMS visits, ED visits and inpatient episodes by their corresponding unit costs. The total cost associated with the Mobile Hospital was derived from actual expenses incurred. The fixed costs of the Mobile Hospital (such as car leasing and equipment costs) were allocated to 5 years. The average daily cost for primary hospital inpatient care was sourced from the city of Espoo. Costs for EMS visits, ED visits and specialised hospital inpatient care were determined using price lists from HUS. These costs include all production costs, including salaries for doctors and nursing staff, inpatient medications, materials, as well as costs related to facilities and equipment. Costs from the 6-month study periods in 2018 and 2019 were extrapolated to provide estimated annual savings for comparison. In the benchmarking analysis, we compared the average number of ED visits per population in 2018 and 2019, as well as the number of emergency admissions. There were no missing values relevant to the analysis.

## Results

Comparing the residents in nursing homes 2018 to 2019, there were no differences in mean age and sex/gender. Top 10 diagnoses for nursing home patients visiting the ED are presented in Appendix 1 in the Supplementary Data section. In the 2018 six-month pre-intervention period, 59.6% of nursing home patients visited the ED (801 ED visits for 1345 nursing home patients). In the 2019 six-month post-intervention period, 46.4% of nursing home patients visited the ED (622 ED visits for 1342 nursing home patients). Descriptive statistics are presented in Table 1.

**Table 1:** ED and specialised hospital ward statistics per 6-month study period

	Pre-2018	Post-2019	*P*-value
Residents living in nursing homes (total *N*)	1345	1342	.95 NS
Sex/gender (percent of female, available from EMS data)	61.7%	63.3%	.38 NS
ED visits (*N*)	801	622	<.001^*^
ED throughput time in hours (mean, SD)	7.79 (5.54)	7.00 (5.55)	<.001^*^
Patients continuing to specialised hospital ward, *N* (%)	178 (22.2%)	162 (26.0%)	.11 NS
Length of stay in specialised hospital ward in days (mean, SD)	3.85 (3.63)	4.57 (4.56)	.15 NS
Mortality (not including terminal care in nursing home)	11 (0.8%)	19 (1.4%)	.14 NS

*N*, number of patients; SD, standard deviation; NS, not statistically significant.

^*^Statistically significant.

In 2019, with the Mobile Hospital intervention in use, several key metrics related to acute care for nursing home residents showed significant changes compared to 2018, when the intervention was not in place. ED throughput times were faster. The number of EMS missions to nursing homes decreased by 16% (from 720 to 604). ED visits decreased by 22% (from 801 to 622). ED readmissions within 24 h remained stable (from 128 to 108). Number of specialised inpatient episodes remained stable (from 178 to 162), while primary hospital inpatient episodes decreased by 38% (from 285 to 178).

**Figure 1:**
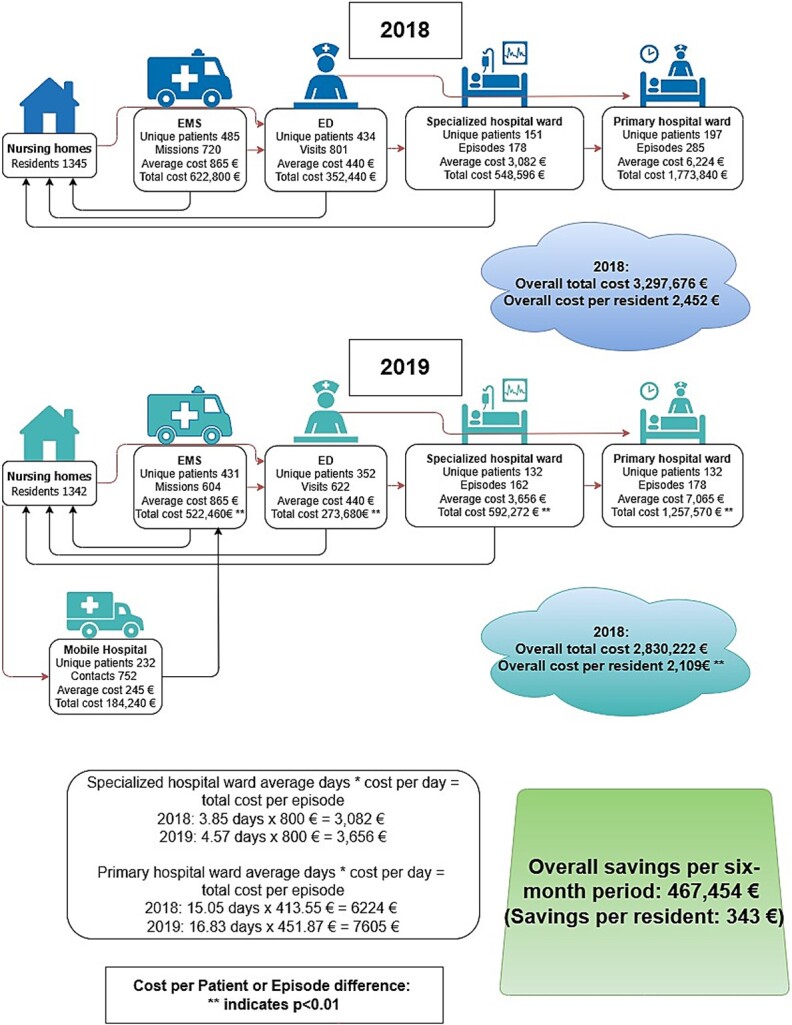
Comparison of 2018 and 2019 six-month study periods: total costs of acute care for nursing home residents.

The cost of the Mobile Hospital service for the 6-month study period was 184 240 euros. In the 2018 six-month study period, before the Mobile Hospital was introduced, the total cost of acute care and subsequent hospitalisation per EMS patient was 6799 euros. During the 2019 six-month study period, with the Mobile Hospital in operation, the total cost of acute care and subsequent hospitalisation per EMS and Mobile Hospital patient decreased to 4269 euros—a cost savings of 2530 euros, representing a 37% reduction. The annual estimated savings per nursing home resident were 686 euros, representing a 14% reduction. The total annual estimated savings were 934 908 euros. Other costs are presented in Figure 1.

### Benchmarking validation data

**Figure 2:**
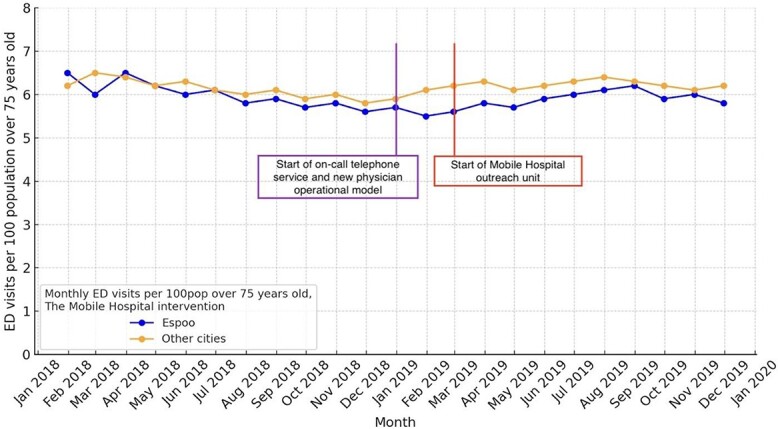
Monthly ED visits per 100pop, the Mobile Hospital intervention.

Our inspection of the parallelism in pre-intervention trends supported the suitability of the benchmarking analysis used for outcome comparisons. The yearly emergency visits per 100 over 75-year-old people decreased (*P* < .01) from 72 (year 2018) to 67 (year 2019) in Espoo as in the same period the average of big cities increased from 70 (year 2018) to 78 (year 2019) (Figure 2). The respective number of acute hospitalisations decreased (*P* < .01) from 27 (2018) to 20 (2019) in Espoo as the average for other cities remained 37 for other cities years 2018 and 2019.

## Discussion

In 2019, with the Mobile Hospital intervention in use, most of the variables related to nursing homes showed significant improvements: ED throughput times, number of EMS missions to nursing homes, ED visits and primary hospital inpatient episodes. These changes led to significant estimated cost savings. The Mobile Hospital’s operating costs over the 6-month study period totalled 184 240 euros. We used data from the 6-month study periods in 2018 and 2019 to estimate annual savings. The estimated annual savings per nursing home resident were 686 euros (14% reduction) and per EMS and Mobile Hospital patient 2530 euros (37% reduction). The total annual estimated savings were 934 908 euros.

To account for potential confounding factors, we performed benchmark-controlled analysis by comparing the available measures to other similar EDs of the biggest cities in Finland: According to nationwide benchmarking data, the ED visits and acute hospitalisations for people >75 years old living in Espoo decreased, contrary to the national trend, which was on the rise. These findings suggest that the Mobile Hospital intervention provided necessary acute care on-site for nursing home residents, which would have otherwise required ED visits. This finding aligns with a recent systematic review [3] and a recent study that seven out of eight nursing home residents could avoid hospitalisation if professional acute evaluation is available [4].

Because of legislative changes and other complicating factors, this study was primarily conducted using a pre–post design as patient-level data were not readily available from other similar EDs. This study, conducted in one Nordic country, may have limited applicability to regions with different acute and older adult care systems. In Espoo, nursing home physicians typically visit monthly, potentially allowing the Mobile Hospital to handle more acute cases than regional models with weekly physician visits. Presumably unlike these physicians, the Mobile Hospital is equipped with POC testing and intravenous medications.

Two simultaneous changes in Espoo were observed after data collection: starting in January 2019, nursing home physician services were recontracted and transitioned to a new operational model. Weekly doctor visits in nursing homes were replaced by weekly phone consultations and monthly on-site visits; more advance care planning was required, and an on-call telephone service for nursing homes became available on weekdays from 8 a.m. to 7 p.m. Although advance care planning alone might reduce EMS missions and ED visits for nursing home residents, the certainty of the evidence remains low [12]. These changes could have partially accounted for the observed outcomes, suggesting that the Mobile Hospital could be described as a multicomponent intervention, somewhat resembling a Canadian model shown to reduce ED visits [13]. On the other hand, an anticipatory effect may have contributed to early changes in care metrics, as healthcare providers may have adjusted practices in preparation for the Mobile Hospital intervention.

Starting in June 2019, the Mobile Hospital also began visiting people in continuous home care, in addition to nursing homes. If this affected the results, the net effect is not expected to favour the Mobile Hospital. Specialised inpatient episodes showed no significant change, which may indicate that especially visits to the ED leading to specialised hospital care were necessary—in other words, not treatable by the Mobile Hospital or EMS in the nursing home.

Exact patient-level durations of primary hospital inpatient episodes were not retrievable; therefore, average length and cost was used. This may have affected the results, although the average lengths of stay were fairly similar in both years. Practically no nursing home residents were transferred straight to hospital (information provided by hospital manager).

The validity of our findings is supported by the comprehensive nationwide benchmarking data of whole population >75 years old used, which allowed for a comparison with other major Finnish cities. We did not exclude patients and used real-world data which in observational study setting promotes the applicability of the findings to practice. However, the pro–post study design could be influenced by unidentified external factors. Additionally, while aggregated national data ensured consistency across metrics, the lack of individual patient data from other regions limited our ability to control for case-mix differences, potentially impacting the precision of cost-effectiveness and clinical outcomes.

We are not aware of any other interventions during the study period that affected ED throughput times. Therefore, the reduced demand likely contributed to shorter throughput times. This suggests that the Mobile Hospital improved timeliness of care not only by providing immediate on-site assessment and treatment but also by reducing wait times for patients who still required ED-based and hospital-based care, thereby enhancing overall acute care system effectiveness.

We were surprised by the small number of neurologic visits. Possible neurologic complaints mostly registered as Internal medicine or General practice in the ED, reflecting the Finnish ED system responsibilities.

No significant mortality changes were noted, but nevertheless mortality amongst nursing home residents is a complex variable, as the primary objective is not merely to extend life at all costs, but rather to enhance the quality of life. This includes the avoidance of painful and burdensome treatments. The analysis of 24-h ED readmission rates showed no significant differences, supporting the idea that the Mobile Hospital does not lead to unnecessary demand on emergency services.

While the optimal study design for the Mobile Hospital system study would be a randomised controlled trial, this benchmarking-controlled trial design still provides valuable insights. Although observational studies introduce some uncertainty regarding potential confounding factors, the absence of other significant changes in other emergency departments during the measurement period enhances the reliability of our findings. Furthermore, the results align with our expectations based on the mechanisms of outreach units, underscoring the importance of this study in advancing healthcare delivery.

Our study provides valuable contributions by focusing on the cost-effectiveness of implementing an outreach service—a topic with limited existing research [3]. The substantial cost savings estimate of 37% reduction per acute care patient, and 14% per nursing home resident, aligns with the cost savings reported in few prior studies on somewhat similar interventions [14–16]. To our knowledge, this is the first benchmarking-controlled trial examining the outreach unit (the Mobile Hospital) to nursing homes. Our study also presents new insights into how the total acute care costs are associated with outreach care services.

The evidence supporting outreach care to nursing homes is growing, and healthcare staff experiences regarding these units are encouraging [17]. We recommend using a stepped-wedge or quasi-controlled trial in future studies to enhance the reliability of conclusions on quality and cost-effectiveness.

## Conclusions

The Mobile Hospital seems to promote care quality by reducing nursing home residents’ ED visits, hospitalisations and overall costs (at least when part of a multicomponent intervention focusing on availability of acute on-site and remote care and advance care planning). More research is needed on the topic, preferably using stepped-wedge cluster randomised trial setting with real costs.

Implications to practice: This study adds to the growing evidence that outreach care to nursing homes is cost-effective in suburban areas with universal healthcare funding, seemingly as a part of other developments to care pathway.

## Abbreviations

ED, emergency department; EMS, Emergency Medical Services; HILMO, National Registry of Hospital Visits and Inpatient episodes; HUS, Helsinki University Hospital; IOM, Institute of Medicine; MD, medical doctor; NS, not statistically significant; POC, point of care; SD, standard deviation; STROBE, Strengthening the Reporting of Observational studies in Epidemiology; THL, Finnish Institute of Health and Welfare.

## Supplementary Material

aa-24-1422-File003__afae287
