# Supplementary material for: Outreach acute care for nursing homes: an observational study on the quality and cost-effectiveness of the Mobile Hospital
Source: Age Ageing. 2025 Jan 7;54(1):afae287. doi: 10.1093/ageing/afae287 (PMC11705071; doi:10.1093/ageing/afae287)
Supplement: aa-24-1422-File003__afae287 [file aa-24-1422-file003__afae287.docx]

**Outreach Acute Care for Nursing Homes: An Observational Study on the quality and cost-effectiveness of the Mobile Hospital**

**Appendices:**

**Appendix 1 Supplementary Table: Number of ED Top 10 Diagnoses (ICD-10)**

**Appendix 2 Supplementary document: STROBE checklist**

**Appendix 1 Supplementary Table: Number of ED Top 10 Diagnoses (ICD-10)**

| **Pre 2018** | **N** | **Post 2019** | **N** |
| --- | --- | --- | --- |
| R53 Malaise and fatigue | 73 | R53 Malaise and fatigue | 39 |
| J18.9 Pneumonia | 40 | J18.9 Pneumonia | 30 |
| S01.0 Open wound of scalp | 24 | S01.0 Open wound of scalp | 28 |
| R07.4 Chest pain | 23 | R07.4 Chest pain | 22 |
| A09 Gastroenteritis | 19 | A49.9 Bacterial infection, unspecified | 18 |
| N10 Pyelonephritis | 18 | I50.9 Heart failure | 16 |
| R06.0 Dyspnea | 18 | R06.0 Dyspnea | 15 |
| I48 Atrial fibrillation or flutter | 17 | N10 Pyelonephritis | 14 |
| I50.9 Heart failure | 17 | R10.4 Other and unspecified abdominal pain | 14 |
| Z43.5 Encounter for attention to cystostomy | 16 | I48 Atrial fibrillation or flutter | 13 |

**Appendix 2 Supplementary document: STROBE checklist**

STROBE Statement—checklist of items that should be included in reports of observational studies

|  | **Item No.** | **Recommendation** | **Page No.** | **Relevant text from manuscript** |
| --- | --- | --- | --- | --- |
| **Title and abstract** | **1** | **(*a*) Indicate the study’s design with a commonly used term in the title or the abstract** | **1** | Observational study |
|  |  | **(*b*) Provide in the abstract an informative and balanced summary of what was done and what was found** | **2** | This study describes the results of the Mobile Hospital intervention to nursing homes in a pre-post study setting with benchmarking validation data.  Methods: We compared Emergency Medical Services (EMS) missions, ED visits, hospitalizations, and their estimated costs from two six-month periods in 2018-2019 (1325 nursing home beds). Benchmarking control data for ED visits was obtained from health records of the 10 largest Finnish cities.  Results: The number of EMS missions to nursing homes decreased by 16% (720 vs 604), ED visits decreased by 22% (801 vs 622), there was no significant difference in specialized inpatient episodes (178 vs 162), and primary hospital inpatient episodes were fewer (285 vs 178, decreased 38%). Annual estimated savings per resident were 686 euros (decreased 14%). Annual estimated total savings were 934,908 euros. In the benchmarking analysis, the number of ED visits and acute hospitalizations among the older population decreased in Espoo, while in the other cities it increased. |
| **Introduction** | | | |  |
| **Background/rationale** | **2** | **Explain the scientific background and rationale for the investigation being reported** | **2-3** | (Please see Background pages 2-3) |
| **Objectives** | **3** | **State specific objectives, including any prespecified hypotheses** | **4** | Our primary objective was to examine the quality and cost-effectiveness of the Mobile Hospital intervention by comparing registry data from 2018 and 2019 six-month study periods. |
| **Methods** | | | |  |
| **Study design** | **4** | **Present key elements of study design early in the paper** | **3-5** | We studied the Mobile Hospital intervention by  1) Analyzing the changes in process, outcomes, and cost measures before and after the intervention (2018 pre-period vs 2019 post period), and  2) Comparing the number of ED visits and acute hospitalizations among people over 75 years in Espoo with the other biggest cities in Finland. |
| **Setting** | **5** | **Describe the setting, locations, and relevant dates, including periods of recruitment, exposure, follow-up, and data collection** | **3-5** | (Please see item 4) |
| **Participants** | **6** | **(*a*) *Cohort study*—Give the eligibility criteria, and the sources and methods of selection of participants. Describe methods of follow-up**  ***Case-control study*—Give the eligibility criteria, and the sources and methods of case ascertainment and control selection. Give the rationale for the choice of cases and controls**  ***Cross-sectional study*—Give the eligibility criteria, and the sources and methods of selection of participants** | **3-5** | Cohort study (Pre-post) with benchmarking |
|  |  | **(*b*) *Cohort study*—For matched studies, give matching criteria and number of exposed and unexposed**  ***Case-control study*—For matched studies, give matching criteria and the number of controls per case** | **-** | (Not applicable) |
| **Variables** | **7** | **Clearly define all outcomes, exposures, predictors, potential confounders, and effect modifiers. Give diagnostic criteria, if applicable** | **3-5** | (Please see item 4 + )  In the pre-post setting the primary outcome measures were the numbers of EMS missions, ED visits, Specialized hospital episodes due to acute reasons = hospitalization from the ED (not included elective episodes) and acute Primary Hospital episodes … We also reviewed the Mobile Hospital missions and total cost. From this data and list prices of EMS missions, ED visits and hospital days we estimated the costs. As a relative safety marker we compared mortality rates and 24-hour ED readmission rates. … We received aggregate inpatient data from primary hospital ward and therefore the length of stay in primary hospital ward is based on average length of stay. |
| **Data sources/ measurement** | **8*** | **For each variable of interest, give sources of data and details of methods of assessment (measurement). Describe comparability of assessment methods if there is more than one group** | **5** | We collected the data from electronic health records. … We received aggregate inpatient data from primary hospital ward and therefore the length of stay in primary hospital ward is based on average length of stay. We collected the Benchmarking validation data from … |
| **Bias** | **9** | **Describe any efforts to address potential sources of bias** | **5** | (Benchmarking) |
| **Study size** | **10** | **Explain how the study size was arrived at** | **-** | (Observational study on regional data) |

**Continued on next page**

| **Quantitative variables** | **11** | **Explain how quantitative variables were handled in the analyses. If applicable, describe which groupings were chosen and why** | **5** | SPSS and Excel were used with Chi Square, t-test and Mann-Whitney U statistical tests as appropriate. We used Large language model ChatGPT 4 (OpenAI) to help proofread and present the results. The fixed costs of the Mobile Hospital (such as car lease and equipment costs) were allocated to five years. We compared the average number of visits per population for years 2018 and 2019 as well as the number of emergency admissions respectively. |
| --- | --- | --- | --- | --- |
| **Statistical methods** | **12** | **(*a*) Describe all statistical methods, including those used to control for confounding** | **5** | (Please see item 11) |
|  |  | **(*b*) Describe any methods used to examine subgroups and interactions** | **-** |  |
|  |  | **(*c*) Explain how missing data were addressed** | **5** | There were no missing values relevant to the analysis. |
|  |  | **(*d*) *Cohort study*—If applicable, explain how loss to follow-up was addressed**  ***Case-control study*—If applicable, explain how matching of cases and controls was addressed**  ***Cross-sectional study*—If applicable, describe analytical methods taking account of sampling strategy** | **-** | (not applicable) |
|  |  | **(*e*) Describe any sensitivity analyses** | **-** |  |
| **Results** | | | | |
| **Participants** | **13*** | **(a) Report numbers of individuals at each stage of study—eg numbers potentially eligible, examined for eligibility, confirmed eligible, included in the study, completing follow-up, and analysed** | **6** | (Please see Table 1.) |
|  |  | **(b) Give reasons for non-participation at each stage** | **-** | (not applicable) |
|  |  | **(c) Consider use of a flow diagram** | **7** | (Please see Figure 1.) |
| **Descriptive data** | **14*** | **(a) Give characteristics of study participants (eg demographic, clinical, social) and information on exposures and potential confounders** | **6** | (Please see Table 1.) |
|  |  | **(b) Indicate number of participants with missing data for each variable of interest** | **-** | (not applicable) |
|  |  | **(c) *Cohort study*—Summarise follow-up time (eg, average and total amount)** | **4** | (Six months in 2018 vs six months in 2019) |
| **Outcome data** | **15*** | ***Cohort study*—Report numbers of outcome events or summary measures over time** | **7** | (Please see Figure 1.) |
|  |  | ***Case-control study—*Report numbers in each exposure category, or summary measures of exposure** |  |  |
|  |  | ***Cross-sectional study—*Report numbers of outcome events or summary measures** |  |  |
| **Main results** | **16** | **(*a*) Give unadjusted estimates and, if applicable, confounder-adjusted estimates and their precision (eg, 95% confidence interval). Make clear which confounders were adjusted for and why they were included** | **6-7** | (Please see Table 1. and Figure 1.) |
|  |  | **(*b*) Report category boundaries when continuous variables were categorized** | **-** | (not applicable) |
|  |  | **(*c*) If relevant, consider translating estimates of relative risk into absolute risk for a meaningful time period** | **-** |  |

**Continued on next page**

| **Other analyses** | **17** | **Report other analyses done—eg analyses of subgroups and interactions, and sensitivity analyses** | **-** |  |
| --- | --- | --- | --- | --- |
| **Discussion** | | | | |
| **Key results** | **18** | **Summarise key results with reference to study objectives** | **8** | In 2019, with the Mobile Hospital intervention in use, most of the variables related to nursing homes showed significant improvements: ED throughput times, number of EMS missions to nursing homes, ED visits and primary hospital inpatient episodes. These changes led to significant cost savings.  To account for potential confounding, we performed benchmark controlling: According to nationwide benchmarking data, the ED visits and acute hospitalizations for people over 75 years old living in Espoo decreased, contrary to the national trend, which was on the rise. |
| **Limitations** | **19** | **Discuss limitations of the study, taking into account sources of potential bias or imprecision. Discuss both direction and magnitude of any potential bias** | **9** | Two simultaneous changes were discovered after the data collection: (physician service change and advance care planning) This could have accounted for the observed changes, and in that case the Mobile Hospital could be described as a multicomponent intervention … From June 2019 the Mobile Hospital started also visiting people in continuous home care in addition to nursing homes, if this would affect the results the net effect is not expected to be in favor of the Mobile Hospital.  … Exact patient-level durations of primary hospital inpatient episodes were not retrievable, therefore an average length and cost was used. This may have affected the results, although the average lengths of stay were fairly similar in both years. |
| **Interpretation** | **20** | **Give a cautious overall interpretation of results considering objectives, limitations, multiplicity of analyses, results from similar studies, and other relevant evidence** | **9** | The Mobile Hospital seems to reduce nursing home residents’ ED visits, hospitalizations and overall costs (at least when part of a multicomponent intervention focusing on availability of acute on-site and remote care and advance care planning). |
| **Generalisability** | **21** | **Discuss the generalisability (external validity) of the study results** | **9** | This study adds to the growing evidence that outreach care to nursing homes is cost-effective in suburban areas with universal healthcare funding, seemingly as a part of other developments to care pathway. |
| **Other information** | |  | | |
| **Funding** | **22** | **Give the source of funding and the role of the funders for the present study and, if applicable, for the original study on which the present article is based** | **1** | (Please see page 1. No conflicts of interest.) |

***Give information separately for cases and controls in case-control studies and, if applicable, for exposed and unexposed groups in cohort and cross-sectional studies.**

**Note: An Explanation and Elaboration article discusses each checklist item and gives methodological background and published examples of transparent reporting. The STROBE checklist is best used in conjunction with this article (freely available on the Web sites of PLoS Medicine at http://www.plosmedicine.org/, Annals of Internal Medicine at http://www.annals.org/, and Epidemiology at http://www.epidem.com/). Information on the STROBE Initiative is available at www.strobe-statement.org.**
